# Quantitative ^1^H-NMR-Metabolomics Reveals Extensive Metabolic Reprogramming and the Effect of the Aquaglyceroporin *FPS1* in Ethanol-Stressed Yeast Cells

**DOI:** 10.1371/journal.pone.0055439

**Published:** 2013-02-08

**Authors:** Artur B. Lourenço, Filipa C. Roque, Miguel C. Teixeira, José R. Ascenso, Isabel Sá-Correia

**Affiliations:** 1 IBB - Institute for Biotechnology and Bioengineering, Centre for Biological and Chemical Engineering, Instituto Superior Técnico, Technical University of Lisbon, Lisboa, Portugal; 2 Centro de Química Estrutural, Instituto Superior Técnico, Technical University of Lisbon, Lisboa, Portugal; University Paris South, France

## Abstract

A metabolomic analysis using high resolution ^1^H NMR spectroscopy coupled with multivariate statistical analysis was used to characterize the alterations in the endo- and exo-metabolome of *S. cerevisiae* BY4741 during the exponential phase of growth in minimal medium supplemented with different ethanol concentrations (0, 2, 4 and 6% v/v). This study provides evidence that supports the notion that ethanol stress induces reductive stress in yeast cells, which, in turn, appears to be counteracted by the increase in the rate of NAD^+^ regenerating bioreactions. Metabolomics data also shows increased intra- and extra-cellular accumulation of most amino acids and TCA cycle intermediates in yeast cells growing under ethanol stress suggesting a state of overflow metabolism in turn of the pyruvate branch-point. Given its previous implication in ethanol stress resistance in yeast, this study also focused on the effect of the expression of the aquaglyceroporin encoded by *FPS1* in the yeast metabolome, in the absence or presence of ethanol stress. The metabolomics data collected herein shows that the deletion of the *FPS1* gene in the absence of ethanol stress partially mimics the effect of ethanol stress in the parental strain. Moreover, the results obtained suggest that the reported action of Fps1 in mediating the passive diffusion of glycerol is a key factor in the maintenance of redox balance, an important feature for ethanol stress resistance, and may interfere with the ability of the yeast cell to accumulate trehalose. Overall, the obtained results corroborate the idea that metabolomic approaches may be crucial tools to understand the function and/or the effect of membrane transporters/porins, such as Fps1, and may be an important tool for the clear-cut design of improved process conditions and more robust yeast strains aiming to optimize industrial fermentation performance.

## Introduction

*Saccharomyces cerevisiae* is very important in winemaking and bio-ethanol production industries [1]. However, the accumulation of ethanol in the culture broth medium during alcoholic fermentation acts as a stress factor which limits the yield of bioethanol production [2], [3] and affects the quality of wines [4], [5], [6]. Therefore, understanding the toxic effects caused by ethanol and the molecular mechanisms triggered by yeast to cope with this stress is of fundamental importance for strain and process development.

Cellular membranes are the primary target of ethanol [3], which, due to its lipophilicity, is incorporated in these structures changing their organization and affecting their function as a semi-permeable barrier and matrix for enzymes. By affecting the plasma membrane function, ethanol inhibits associated transport processes, the maintenance of the transmembrane electrochemical potential and the plasma membrane function as a selective barrier, leading to intracellular acidification [3], [7], [8]. Ethanol has also been shown to influence cell metabolism, particularly affecting the activity of crucial glycolytic enzymes [9], [10]. Although usually repressed in the presence of glucose, the first step in the metabolism of alcohol is the oxidation of ethanol to acetaldehyde catalyzed by alcohol dehydrogenase, containing the coenzyme NAD^+^. Acetaldehyde is further oxidized to acetic acid and finally CO_2_ and water through the citric acid cycle. In response to ethanol stress, yeast stimulates the activity of the plasma membrane H^+^-ATPase to counteract intracellular acidification [11], [12], [13] and promotes the remodelling of plasma membrane composition by changing its levels of unsaturated fatty acids and ergosterol [11], [14], and of the cell wall composition and structure [15], [16]. Interestingly, yeast tolerance to ethanol has recently been shown to depend on the expression of the ABC multidrug efflux pump Pdr18 [17], involved in ergosterol incorporation in the yeast plasma membrane [18]. Despite the efforts made hitherto, the repercussion of ethanol stress in yeast cells is still not completely understood.

In recent years, an increase in the exploitation of different global approaches has been observed, particularly transcriptomic and chemogenomic approaches, to gain greater insight into the yeast response and tolerance to ethanol stress, at a systems level. Several works have used transcriptomics to analyse the effect that short-term sub-lethal ethanol exposure has in *S. cerevisiae* [19], [20], [21]. These studies suggested the importance of biological processes associated to cell energetics, transport mechanisms, lipid metabolism, trehalose metabolism, glycolysis and tricarboxylic acid cycle (TCA cycle) in the context of yeast response to ethanol stress [22]. In parallel, many functional genomic screens were performed to identify determinants of resistance to ethanol stress [16], [23], [24], [25], [26], [27]. One of these screens, in which 254 yeast genes were identified as being required for maximal tolerance to ethanol, was performed in our laboratory [16]. Among these determinants of resistance, particular attention was given to the *FPS1* gene, encoding a plasma membrane aquaglyceroporin [28], that was found to decrease intracellular accumulation of radiolabelled ethanol [16]. The activity of Fps1 is determined by the osmolarity of the environment. It was proposed that under hyperosmotic shock Fps1 activity is reduced leading to glycerol accumulation, whereas upon shifting back to hypo-osmotic conditions the Fps1 channel opens to release glycerol and thus relieve turgor pressure [28], [29]. Although Fps1 natural substrate seems to be glycerol, Fps1 appears to facilitate the diffusion of toxic compounds across the yeast plasma membrane, including the trivalent metalloids arsenite and antimonite [30], acetic acid [31] and boron [32]. On the other hand, Fps1 deletion has been shown to increase yeast susceptibility to numerous chemical stress inducers, including, according to the SGD compilation, tunicamycin, dithiotreitol, mercaptoethanol, telluride, cadmium chloride, 1-propanol, ethanol, sulfometuron methyl, cycloheximide, caffeine and rapamycin (Saccharomyces Genome Database – www.yeastgenome.com). It does not seem likely, however, that Fps1 plays a direct role in facilitating the diffusion of all these chemicals across the plasma membrane. Significantly, *FPS1* deletion was shown to decrease ergosterol concentration in the yeast plasma membrane [33], an effect likely to interfere with all transmembrane transport systems. Given the role of this aquaglyceroporin in the control of the intracellular content of glycerol [29], we further hypothesized that Fps1 may have a broader effect in the yeast metabolome, which, in turn, may indirectly affect multiple chemical stress resistance.

In this work, we performed a metabolomic analysis to unravel the alterations in the yeast metabolome due to ethanol stress, and to clarify the importance of Fps1 under these conditions. Two previous studies using GC-MS-based metabolomic approaches focused on the yeast response to ethanol-induced stress at the endo-metabolome level, when cultivated in YPD rich media [34], [35]. Although the final aim of metabolomics is to identify and quantify all the intra- and extra-cellular metabolites of an organism, the characteristics of the different metabolites make the output of any metabolic approach tightly dependent on the metabolomic strategy employed [36]. Indeed, each metabolic strategy only gives a part and complementary perspective of the full metabolome picture. We are, to the best of our knowledge, the first to explore a ^1^H-NMR-based metabolomic approach coupled with a multivariate statistical analysis to study the alterations both in the exo- and endo-metabolome of yeast cells in the presence of increasing concentrations of ethanol (up to 6% v/v), revealing changes in the relative concentration of 35 metabolites, 40% of which had not been previously identified in this context. This study was undertaken in a controlled minimal growth medium, more suitable to analyse differences at the exo-metabolome level. This metabolomic analysis further examined the effect of the deletion of the *FPS1* gene in the yeast metabolome both in the absence and presence of ethanol stress (2% v/v), thus shedding light on the pleiotropic effect of the expression of this aquaglyceroporin.

## Materials and Methods

### 1. Strains, Growth Media and Growth Conditions

*Saccharomyces cerevisiae* BY4741 (*MATa his3Δ1 leu2Δ0 met15Δ0 ura3Δ0*) parental strain and the mutant *fps1Δ* with the *FPS1* gene deleted, were obtained from the Euroscarf collection. Cells were batch-cultured at 30°C, with orbital agitation (250 rpm) in minimal growth medium MM4. This medium contained (per liter): 1.7 g yeast nitrogen base without amino acids or NH_4_^+^ (Difco), 20 g glucose (Merck), 2.65 g (NH_4_)_2_SO_4_ (Merck), 20 mg methionine (Merck), 20 mg histidine (Merck), 60 mg leucine (Sigma) and 20 mg uracil (Sigma). To examine the effect of ethanol, MM4 medium was supplemented with different ethanol concentrations (2, 4 and 6% v/v of ethanol). Growth curves were followed by measuring culture optical density (OD_600 nm_). Cells used to prepare the inoculum were cultivated in the absence of ethanol until mid-exponential phase at a standardized OD_600 nm_ = 0.5±0.01. These cells were used to inoculate the different media to obtain an initial OD_600 nm_ = 0.2±0.01.

### 2. Metabolome Profiling

#### 2.1 Cell sampling, cold methanol quenching and metabolite extraction

*Saccharomyces cerevisiae* BY4741 parental strain was cultivated in the absence or presence of different ethanol concentrations (2, 4 and 6% v/v) and harvested (120 mg of biomass) during the exponential growth phase when the culture OD_600 nm_ reached values of 1.00±0.05. In the case of the deletion mutant *fps1Δ*, cell samples were collected at the same phase of growth (OD_600 nm_ = 1.00±0.05) either in the absence or presence of 2% v/v of ethanol. Sample preparation for the endo-metabolome analysis was done as before [37]. In order to capture snapshots as accurate as possible of the endo-metabolome, cell metabolism was quenched using a previously described method [38]. One volume of cell culture was immediately added in three volumes of methanol cold solution (60% v/v; T<<−50°C). Cells were pelleted in a centrifuge cooled to −20°C (2,500×g for 6 min). Pellets were washed three times. Eight ml of the methanol cold solution (T<<−50°C) were added to the pellets, vortexed, and pelleted again in a centrifuge cooled to −20°C (2,500×g for 3 min). After each centrifugation step, samples were checked for their temperature using a thermometer (−35°C to +50°C). During quenching and subsequent washing steps, the temperature of the mixture was always below −20°C. Metabolites were extracted based on the method described by Gonzalez *et al*. [39]. Two ml of glass beads (0.4–0.6 mm diameter) and 4 ml of ethanol (75% v/v) were added to the cell pellets, the mixture was then vortexed for 30 s, heated for 3 min to 80°C and vortexed again for 30 s. The supernatant was decanted and the extraction step repeated a second time with 2 ml of ethanol (75% v/v). The supernatants were mixed, cleared by centrifugation (−20°C; 16,000×g; 10 min) and dried under vacuum. For the exo-metabolome analysis, 2 ml of the cell cultures were collected, centrifuged (2,500×g for 3 min) and the supernatant kept in the freezer (−80°C) until NMR data acquisition.

#### 2.2 NMR data acquisition, processing and resonance assignments

For the endo-metabolome analysis, the dried extracts were dissolved in 1 ml of buffer (0.1 M phosphate buffer pH 7.00, in D_2_O, containing 0.1 mM of trimethylsilyl-2,2,3,3-tetradeuteropropionate (d4-TSP)) into 5 mm diameter NMR tubes. The samples were analysed using a 500 MHz Avance III Bruker spectrometer, equipped with a 5 mm inverse probe at 296 K. 1D proton spectra were acquired using a standard NOESY pulse sequence with pre-saturation for water suppression (spectrum width 12 ppm, 32 K data points, relaxation delay 3 s, mixing time 150 ms and 512 scans per FID). For the exo-metabolome analysis, one part of buffered D_2_O was added to three parts of sample (to a final concentration of 0.1 M phosphate buffer pH 7.00 and 0.1 mM of trimethylsilyl-2,2,3,3-tetradeuteropropionate (d4-TSP)), and when necessary the pH was corrected. The proton spectra were collected as before and each spectrum is the result of 128 scans.

All proton spectra were manually phased, baseline corrected and referenced to d4-TSP (δ 0.00 ppm) using Mnova 5.3 for NMR data processing. For metabolite identification purpose 2D J-resolved (128×8 K data points, relaxation delay of 2 s, 128 transients per FID and a spectral width of 62.5 Hz in the 2^nd^ dimension), Total Correlation Spectroscopy (TOCSY; 512×2 K data points, relaxation delay of 2 s, mixing time of 60 ms and 64 transients per FID) and Heteronuclear Single Quantum Coherence (HSQC; 512×2 K data points, relaxation delay of 1.5 s, 64 transients per FID and a spectral width of 174 ppm in indirect dimension) spectra were collected for representative samples.

#### 2.3 Multivariate data analysis

For the endo-metabolome analysis, the spectra regions corresponding to residual water, methanol and ethanol were excluded. Since the total intensity of each spectrum varied markedly with the ethanol concentration present in the culture medium, normalization to the spectrum total intensity is not the best option for data normalization [40]. Therefore, the spectral data were normalized using multiple internal regions of the spectrum (5.000–5.185 ppm, 5.260–6.890 ppm, 6.935–7.075 ppm, 7.165–7.180 ppm, 7.230–7.295 ppm, 7.480–7.895 ppm and 7.985–9.650 ppm). The sum of these internal regions has the same average value (six biological replicates) in all conditions tested and within each condition its intensity is highly correlated with the spectrum total intensity. Unless stated otherwise, the relative abundances of the endo-metabolome data presented herein are always referred to the normalization procedure using these multiple internal regions of the spectrum. Each spectrum was integrated over a series of fixed width bins (0.005 ppm) using Mnova v5.3 features. All spectra were put in a data table form in an ASCI format file and imported into the software SIMCA-P12^+^ for multivariate data analysis. In SIMCA-P12^+^ the data was pre-processed using Pareto scaling. For the exo-metabolome analysis, a more target analysis was used. The spectral data were normalized using the reference peak (TSP). Each metabolite characteristic region (bins with variable width) was integrated using Mnova v5.3 features. The relative abundances of the exo-metabolome data presented within this paper are always referred to the normalization procedure using the reference peak (TSP). All spectral data were put in a data table form in an ASCI format file and imported into the software SIMCA-P12^+^ for multivariate data analysis. In SIMCA-P12^+^ the data was pre-processed using UV scaling. Both the endo- and exo-metabolome data were analysed using Principal Component Analysis (PCA) and Orthogonal Projection on Latent Structures Discriminant Analysis (O-PLS-DA). Unsupervised methods like PCA give an overview of the data and cluster the observations, while supervised methods based on prior information are used to better discriminate between clusters. R^2^ and Q^2^ values were considered as measures of goodness of model and the model robustness, respectively. R^2^ is the fraction of variance explained by a component, and cross validation of this component provides Q^2^, which describes the fraction of the total variation predicted by a component. The value of Q^2^ ranges from 0 to 1 and typically a Q^2^ value greater than 0.4 is considered a good model, and those with Q^2^ values over 0.7 are robust. Interpretation of each supervised model was based on the score plot, the loading plot and the variable importance in the projection plot (VIP). Variables lying in the same region in the loading plot as to the observations in the score plot are enriched in those observations. A measure of the degree to which a particular variable explains cluster membership is obtained with the VIP plot (with confidence intervals derived from jack knifing routine). For a given variable a VIP value above 1 indicates that it is important for class membership.

#### 2.4 Identification of metabolic pathways from metabolic data

MetaboAnalyst [41] was used to identify metabolic pathways more likely associated with the metabolic alterations induced by ethanol stress. A file, in the appropriate format, containing a measure of the concentration (characteristic bin) of all identified metabolites in the endo-metabolome profiles was uploaded to the Pathway analysis (http://www.metaboanalyst.ca/). The dataset used correspond to the data from *S. cerevisiae* BY4741 parental strain in the presence of consecutive concentrations ethanol and from the comparison between *S. cerevisiae* BY4741 parental strain and the derived *fps1Δ* deletion mutant, either in the absence and presence ethanol stress. All metabolites were scaled to UV. *S. cerevisiae* pathway library was selected, and the default options in the pathway analysis algorithms check box were selected, Global test for Pathway enrichment analysis and Relative-betweeness centrality for Pathway topology analysis.

## Results

### 1. Effect of Ethanol on the Growth Curve of *S. cerevisiae* BY4741 and *fps1Δ* Deletion Mutant

**Figure 1:**
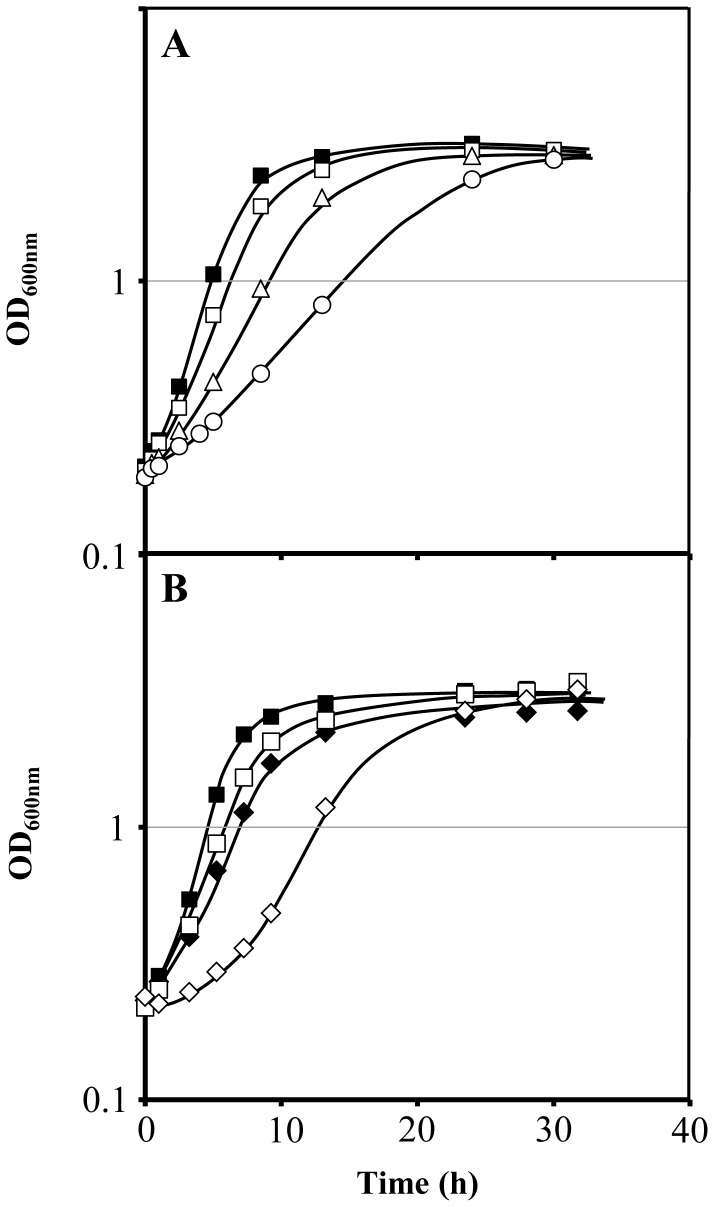
Growth curves of *S. cerevisiae* BY4741 parental strain and *fps1Δ* deletion mutant under ethanol stress. (**A**) Comparison of the growth curves of *S. cerevisiae* BY4741 in the absence (▪) or presence of 2% v/v (□), 4% v/v (Δ) and 6% v/v (○) of ethanol. (**B**) Comparison of the growth curves of *S. cerevisiae* BY4741 (▪, □) and the corresponding deletion mutant *fps1Δ* (♦, ◊) in the absence (closed symbols) or presence of 2% v/v of ethanol (open symbols).

The growth curves of *Saccharomyces cerevisiae* BY4741 parental strain in minimal medium supplemented with different ethanol concentrations (2, 4 and 6% v/v) indicate a dose-dependent reduction of the maximum specific growth rate (Figure 1). Compared to the parental strain, the *fps1Δ* mutant strain, with the *FPS1* gene deleted, exhibited lower maximum specific growth rate even in the absence of ethanol supplementation of the growth medium. In the presence of a low concentration of ethanol (2% v/v), which induces only a slight inhibitory effect on the growth rate of the parental strain, the growth of the *fps1Δ* deletion mutant was drastically affected displaying an initial period of 3 h of growth latency and a reduced maximum specific growth rate (Figure 1).

### 2. Metabolic Characterization of *S. cerevisiae* BY4741 Cells in the Exponential Phase of Growth with Increasing Ethanol Concentrations

The ^1^H NMR spectra from yeast cell extracts (endo-metabolome) and from the culture broth medium (exo-metabolome) (Figure S1) obtained in the exponential phase of growth (OD_600 nm_ of approximately 1.0), either in the presence (2, 4 and 6% v/v) or absence of exogenously added ethanol, enabled the detection of hundreds of resonances. Despite the fact that many of these resonances remain unassigned, some of which exhibiting marked changes, a wide range of classes of biomolecules were identified, including amino acids (L-alanine, L-arginine, L-aspartic and L-glutamic acids), cofactors (NADH, NAD^+^ and NADP^+^), osmolytes (glycerol and trehalose), organic acids (acetic, fumaric and pyruvic acids) and metabolites associated with energy metabolism (ATP, ADP and AMP). Metabolite identification was done by a careful analysis of the chemical shifts, intensities, J couplings and multiplicities of the metabolites peaks present in the 1D proton spectra, complemented with the information from J-resolved, TOCSY and HSQC spectra, and data gathered in different databases, as described before [37]. Indeed, it was possible to identify over 45 metabolites (Figure S1; Tables S1 and S2). All the assigned metabolites, except acetaldehyde and orotidine, were confirmed by spiking with authentic standards.

#### 2.1 Alterations in the endo- and exo-metabolome of *S. cerevisiae* BY4741 in the presence of increasing ethanol concentrations

**Figure 2:**
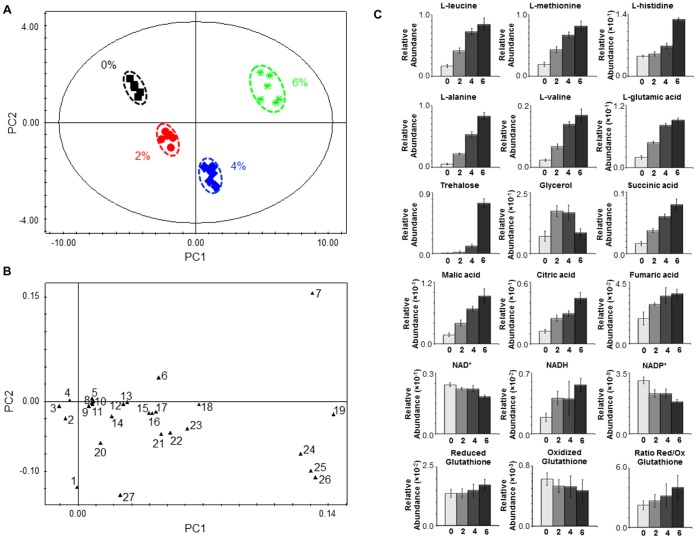
Endo-metabolome analysis of *S. cerevisiae* BY4741 parental strain under ethanol stress. Yeast cells were harvested during the exponential phase of growth (OD_600 nm_ = 1.0) in the same basal medium and supplemented with different ethanol concentrations (% v/v). (**A**) 2D score plot displaying the space formed by the two first principal components (R^2^X for PC1 equal to 0.792 and R^2^X for PC2 equal to 0.129), (**B**) 2D loading plot displaying the space formed by the two first principal components and presenting the characteristic bins for a set of metabolites and (**C**) Relative abundance of different metabolites in the endo-metabolome of *S. cerevisiae* BY4741 cells harvested during the exponential phase of growth (OD_600 nm_ = 1.0) in the presence of different ethanol concentrations (0, 2, 4 and 6% v/v). Each plot displays the variation in the relative abundance of a representative bin in the spectrum for each amino acid. Experimental values are means of six independent experiments with standard deviation error bars. Key: *1* Glycerol, *2* L-arginine, *3* NAD^+^, *4* NADP^+^, *5* L-tyrosine, *6* L-histidine, *7* Trehalose, *8* NADH, *9* Fumaric acid, *10* L-proline, *11* L-phenylalanine, *12* L-asparagine, *13* Citric acid, *14* L-serine, *15* Succininic acid, *16* L-aspartic acid, *17* Malic acid, *18* L-glutamine, *19* L-alanine, *20* L-lysine, *21* L-glycine, *22* L-threonine, *23* L-valine, *24* L-methionine, *25* L-leucine, *26* L-glutamic acid and *27* L-ornithine.

**Figure 3:**
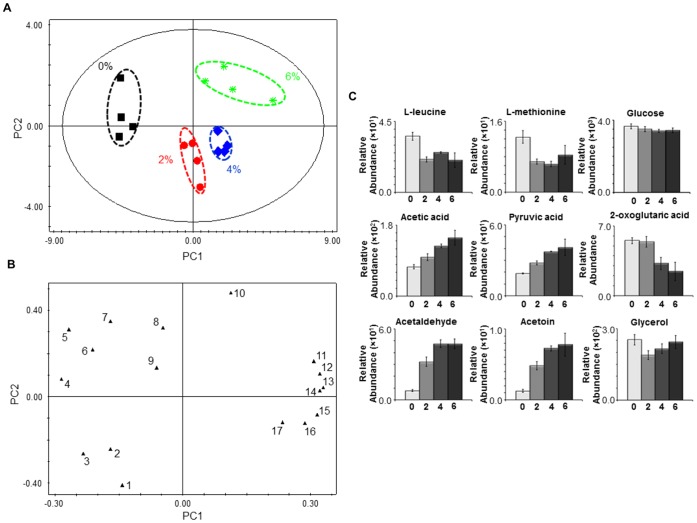
Exo-metabolome analysis of *S. cerevisiae* BY4741 parental strain under ethanol stress. Samples were collected during the exponential phase of growth (OD_600 nm_ = 1.0) in the same basal medium supplemented with different ethanol concentrations (% v/v). (**A**) 2D score plot displaying the space formed by the two first principal components (R^2^X for PC1 equal to 0.522 and R^2^X for PC2 equal to 0.166), (**B**) 2D loading plot displaying the space formed by the two first principal components and presenting the characteristic bins for a set of metabolites and (**C**) relative abundance of different metabolites in the exo-metabolome of *S. cerevisiae* BY4741 cells during the exponential phase of growth (OD_600 nm_ = 1.0) in the presence of different ethanol concentrations (0, 2, 4 and 6% v/v). Experimental values are means of four independent experiments with standard deviation error bars. Key: *1* Orotic acid, *2* Succininc acid, *3* 2-Oxoglutaric acid, *4* L-leucine, *5* L-methionine, *6* Glucose, *7* L-histidine, *8* Glycerol, *9* Formic acid, *10* Uracil, *11* Acetic acid, *12* Pyruvic acid, *13* Acetoin, *14* Acetaldehyde, *15* Lactic acid, *16* L-alanine and *17* Fumaric acid.

The modelling of the endo-metabolome data using Principal Component Analysis (PCA) generated a model with four principal components (PCs) (R^2^X = 0.955 and Q^2^ = 0.928). Figure 2 A shows that the different biological replicates are grouped in four distinct clusters, corresponding to the four different growth conditions (absence and presence of 2, 4 or 6% v/v of ethanol). The analysis of the intracellular metabolic profiles using Orthogonal-Partial Least Squares-Discriminant Analysis (O-PLS-DA) (Table S5), and in accordance with the PCA modelling, clearly distinguished the different conditions under study. The PCA modelling of *S. cerevisiae* BY4741 exo-metabolome data (R^2^X = 0.689 and Q^2^ = 0.432), collected during the exponential phase of growth in the presence of increasing ethanol concentrations, also allows to clearly distinguish the four growth conditions under study (Figure 3 A and B). The analysis of the loading plots (Figures 2 B and 3 B) indicates which metabolites were found to contribute more for cluster separation. The metabolites trehalose, L-alanine, L-methionine, L-leucine and L-glutamic were found to be major contributors for endo-metabolome cluster separation (Figure 2 B).

Using Pathway analysis from MetaboAnalyst (see Materials and methods 2.2.4), we obtained an indication of the metabolic pathways that may be more relevant in the context of ethanol stress. Although this tool is extremely valuable to do an overall analysis of metabolic data, it should be stressed that this analysis is biased to the metabolic pathways containing metabolites covered by the selected metabolomic strategy. Based on the endo-metabolome data of *S. cerevisiae* BY4741 in the absence or presence of 2, 4, and 6% v/v of ethanol, we searched for metabolic pathways with highest scores from the Pathway topology analysis (impact value) and Pathway enrichment analysis (-log p). *Alanine, aspartate and glutamate metabolism* (13.328), *Glutathione metabolism* (13.933), *Glycine, serine and threonine metabolism* (13.087), *Arginine and proline metabolism* (16.211), *Starch and sucrose metabolism* (14.089), *Glycerolipid metabolism* (9.7168), *Nitrogen metabolism* (13.514), *Glyoxylate and dicarboxylate metabolism* (13.542), and *Citrate cycle (TCA cycle)* (13.361) are some of the metabolic pathways, ordered by impact factor with each -log p in brackets, pointed out by this analysis.

Analysing the data in more detail, an increase in amino acid content in yeast cells growing exponentially in the presence of increasing concentrations of ethanol was registered, both for the more abundant (e.g. L-glutamic acid, L-leucine and L-alanine) and in the less abundant (e.g. L-tyrosine, L-proline and L-phenylalanine) amino acids (Figure 2 C; Tables S3 and S4). Exceptions to this general trait are the profiles of L-ornithine, L-lysine and L-arginine (Tables S3 and S4). Significantly, the exometabolome data show that the abundance of L-leucine and L-methionine in the culture broth medium decreases in the same conditions (Figure 3 C; Tables S6 and S7). Alterations in the content of other metabolites were also observed (Figure 2 C; Tables S3 and S4). The content of NAD^+^ decreases while the content of NADH increases with increasing ethanol concentrations (Figure 2 C). Similarly to the observed for NAD^+^, the NADP^+^ content decreases with increasing ethanol concentrations (Figure 2 C). A slight alteration in the oxidized/reduced glutathione ratio was also observed, with the content of the oxidized glutathione slightly decreasing and of the reduced glutathione slightly increasing with increasing ethanol concentrations (Figure 2 C). Of all alterations identified, the most striking occurred in the content of the osmolyte trehalose that increases markedly with increasing ethanol concentrations (Figure 2 C). On the other hand, the glycerol content was higher in the presence of 2 and 4% v/v of ethanol than in absence or presence of 6% v/v of ethanol (Figure 2 C). The TCA cycle associated metabolites succinic, malic, citric and fumaric acids also exhibit a dose-dependent increase in yeast cells growing under ethanol stress (Figure 2 C). A metabolite-to-metabolite correlation analysis of the endo-metabolome data (Figure S2) suggested a link between amino acid and TCA-associated metabolite concentration changes under ethanol stress. Simultaneously, a lower accumulation of 2-oxoglutaric acid and a higher accumulation of acetic and pyruvic acids, acetaldehyde and acetoin were registered in the culture broth of yeast cells growing exponential under ethanol stress (Figure 3 C).

#### 2.2 Effect of *FPS1* expression in the endo- and exo-metabolome of yeast cells in the presence or absence of ethanol stress

**Figure 4:**
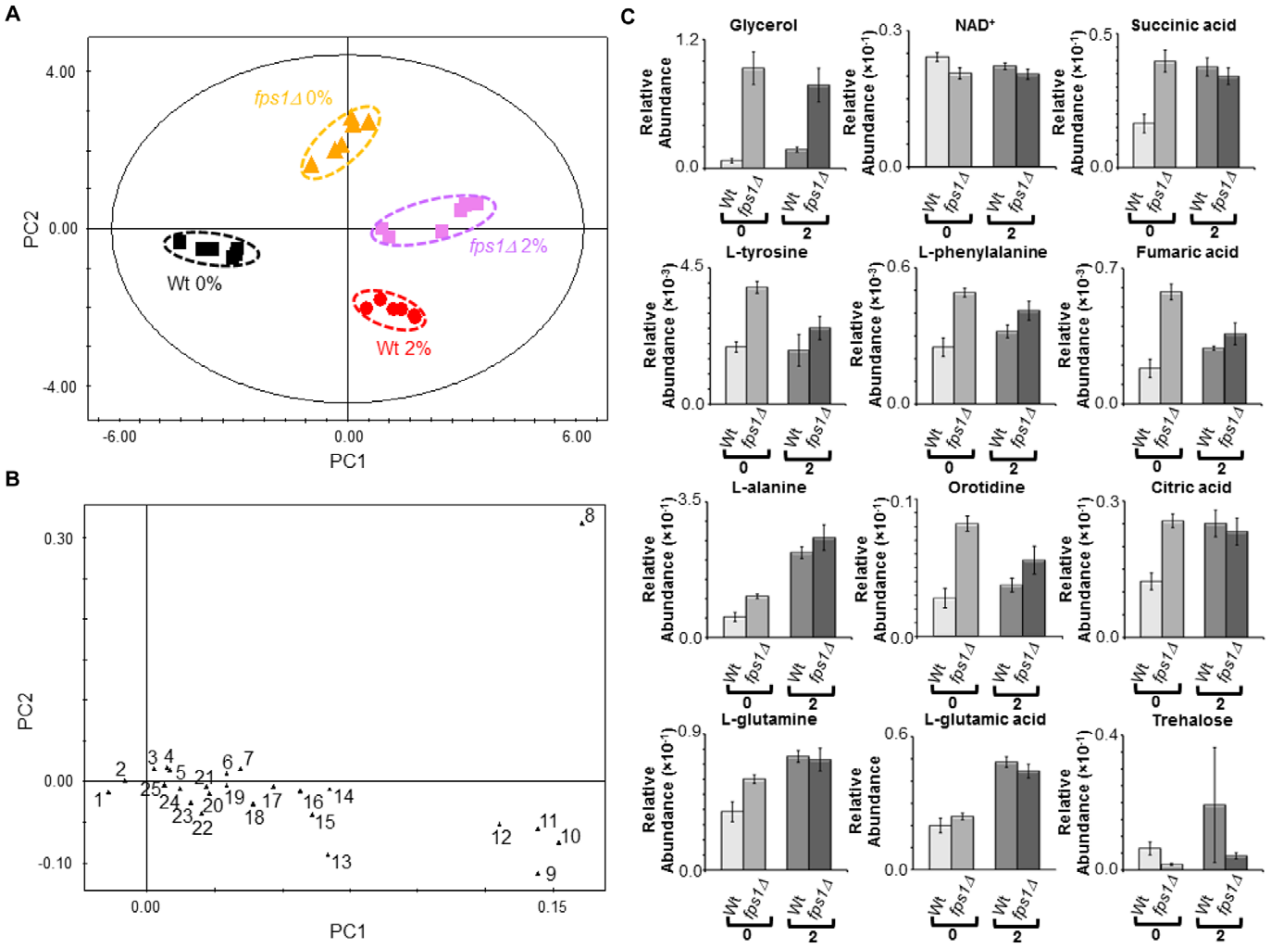
Comparison between the endo-metabolome of BY4741 parental strain and of *fps1Δ* deletion mutant. Yeast cells were harvested during the exponential phase of growth (OD_600 nm_ = 1.0) in the same basal medium supplemented with 0 or 2% v/v of ethanol. (**A**) 2D score plot displaying the space formed by the two first principal components (R^2^X for PC1 equal to 0.525 and R^2^X for PC2 equal to 0.321), (**B**) 2D loading plot displaying the space formed by the two first principal components (with characteristic bins for a set of metabolites) and (**C**) relative abundances of different metabolites in the endo-metabolome of the parental strain and the *fps1Δ* deletion strain during the exponential growth phase (OD_600 nm_ = 1.0) in the presence of different ethanol concentrations (0 and 2% v/v). Experimental values are means of six independent experiments with standard deviation error bars. Key: *1 *NAD^+^, *2 *NADP^+^, *3* L-tyrosine, *4* Fumaric acid, *5* L-phenylalanine, *6* Citric acid, *7* Succininic acid, *8* Glycerol, *9* L-glutamic acid, *10* L-leucine, *11* L-methionine, 12 L-alanine, *13 *L-ornithine, *14* L-threonine, *15* L-valine,*16* L-glutamine, *17 *L-aspartic acid, *18* L-malic acid, *19* L-histidine, *20* L-serine, *21* L-asparagine, *22* L-lysine, *23* Thiamine, *24* NADH and *25* L-proline.

**Figure 5:**
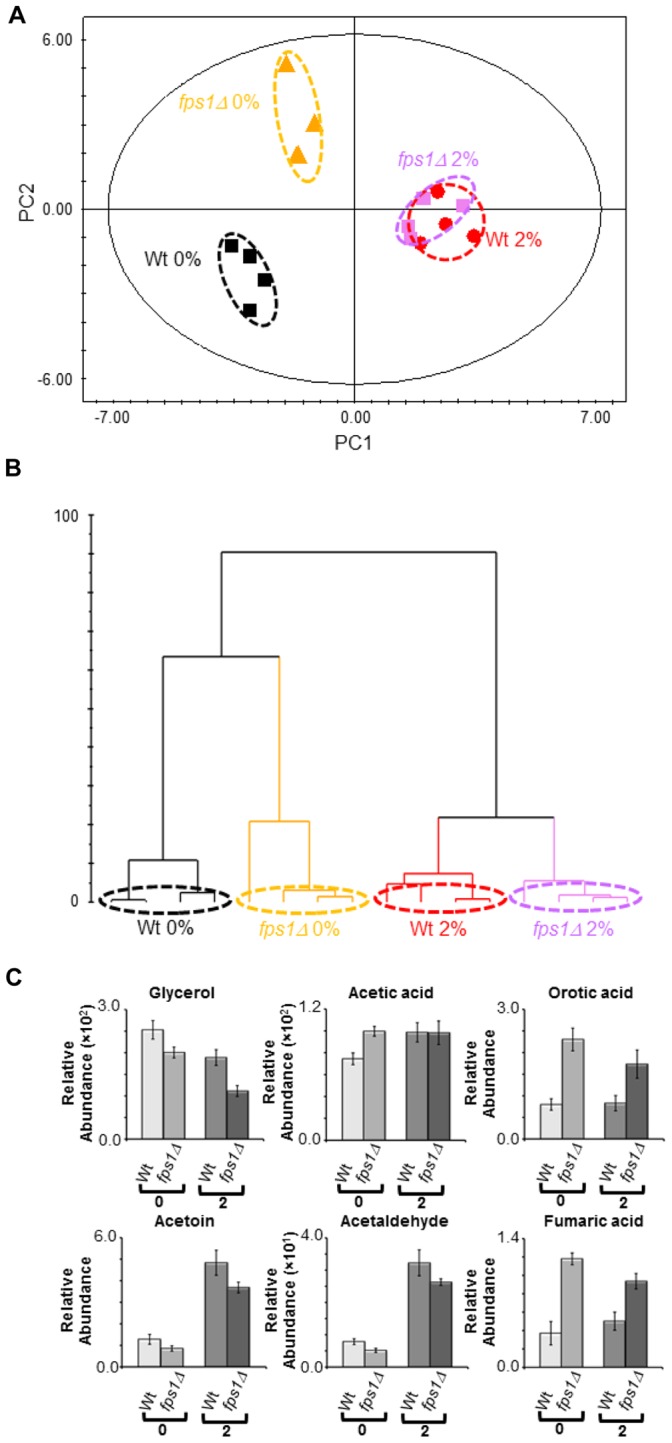
Comparison between the exo-metabolome of BY4741 parental strain and of *fps1Δ* deletion mutant. Samples were collected during the exponential phase of growth (OD_600 nm_ = 1.0) in the same basal medium supplemented with 0 or 2% v/v of ethanol. (**A**) 2D score plot displaying the space formed by the two first principal components (R^2^X for PC1 equal to 0.375 and R^2^X for PC2 equal to 0.284), (**B**) dendrogram based on the PCA scores (ward clustering distance measure) considering all principal components of the model and (**C**) relative abundances of different metabolites in the exo-metabolome of the parental strain and the *fps1Δ* deletion mutant during the exponential growth phase (OD_600 nm_ = 1.0) in the presence of different ethanol concentrations (0 and 2% v/v). Experimental values are means of four independent experiments with standard deviation error bars.

The intracellular metabolic profiles from the *fps1Δ* mutant strain and the parental strain cells harvested in the exponential phase of growth (OD_600 nm_ of approximately 1.0) either in the presence (2% v/v) or absence of exogenously added ethanol were compared using a multivariate statistical analysis. The PCA model (two PCs, R^2^X = 0.846 and Q^2^ = 0.819) clearly distinguishes between both the growth conditions and the yeast strains (Figure 4). The analysis of the score plot (Figure 4 A) indicates a more pronounced difference between the two strains in the absence than in the presence of ethanol stress. The O-PLS-DA modelling (Table S8) pointed the metabolites glycerol, L-leucine and L-methionine, and glycerol, L-glutamic acid and L-alanine as major contributors for strain differentiation, either in the absence or presence of ethanol stress (2% v/v), respectively. The analysis of the exo-metabolome data, collected during the exponential phase of growth (OD_600 nm_ of approximately 1.0) either in the presence (2% v/v) or absence of exogenously added ethanol, gave clustering results similar to the endo-metabolome analysis. The PCA model (five PCs, R^2^X = 0.929 and Q^2^ = 0.467) clearly distinguishes between both the growth conditions and the two yeast strains (Figure 5 A and B). In particular, the growth conditions are distinguishable across the PC1 (Figure 5 A). The metabolic differences between yeast strains are more pronounced in the absence than in the presence of ethanol (Figure 5 B). Several metabolites contribute for strain differentiation (Figure 5 C; Tables S6, S10, S11 and S12).

Pathway analysis pointed out the metabolic pathways *Starch and sucrose metabolism, Glycerolipid metabolism, Nicotinate and nicotinamide metabolism, Nitrogen metabolism, Citrate cycle (TCA cycle)*, and *Phenylalanine, tyrosine and tryptophan biosynthesis* as being possibly linked to the effect of *FPS1* expression in the endo-metabolome of yeast cells in the presence or absence of ethanol stress.

The intracellular content of glycerol was the most striking change registered both in the absence or presence of ethanol stress, being much higher in the *fps1Δ* mutant strain than in the parental strain (Figure 4 C). This is consistent with the observed decrease in glycerol accumulation in the extracellular medium, registered for *fps1Δ* cells (Figure 5 C). Overall metabolite abundances suggest that the deletion of *FPS1* in the absence of ethanol stress has, although in a different magnitude, a similar effect to ethanol stress exposure in the parental strain (Figure 4 C; Tables S4 and S9). In particular, the *fps1Δ* mutant strain has a lower content of NAD^+^ than the parental strain, both in the presence or absence of ethanol stress. In the absence of ethanol supplementation, the content of succininc, citric and fumaric acids is higher in the *fps1Δ* mutant strain than in the parental strain. The intracellular and extracellular content of fumaric acid is also higher in the *fps1Δ* mutant strain than in the parental strain in the presence of ethanol stress (Figures 4 C and 5 C). Similarly, the content of L-phenylalanine and L-tyrosine is higher in the *fps1Δ* mutant strain than in the parental strain both in the absence and presence of ethanol supplementation (Figure 4 C). Differences were also observed in the content of other amino acids (e.g. L-alanine, L-glutamic acid and L-glutamine) either in the absence or presence of exogenously added ethanol (Figure 4 C). Furthermore, the content of intracellular orotidine (Figure 4 C) and extracellular orotic acid (Figure 5 C) was also found to be higher in the *fps1Δ* mutant strain than in the parental strain in both conditions tested. On the other hand, the content of trehalose was found to be higher in the parental strain than in the *fps1Δ* mutant strain both in the absence and presence of ethanol stress (Figure 4 C), although in this last case, due to the variability registered in the parental strain, it did not pass the statistical test (P-value 1.05×10^−1^).

Finally, the *fps1Δ* deletion mutant was found to excrete lower amounts of acetaldehyde and acetoin in the culture broth medium than the parental strain in both conditions tested (Figure 5 C), while, in the absence of exogenously added ethanol, the *fps1Δ* strain exo-metabolome exhibits 23% and 34% higher levels of ethanol and acetic acid (Figure 5 C) than that of the parental strain, respectively.

## Discussion

In this study the changes occurring in the endo- and exo-metabolome of yeast cells that allow them to grow exponentially in the presence of inhibitory concentrations of ethanol were characterized by high resolution ^1^H NMR spectroscopy. Previously, Ding *et al.* and Li *et al.* [34], [35] used GC-MS-based metabolomic approaches to study the yeast response to ethanol-induced stress at the endo-metabolome level. Based on the metabolites that were reported to be, simultaneously, detected and identified in those studies, the work herein have significantly increased the metabolic coverage at the endo-metabolome level (30% increase), emphasizing the importance of complementary strategies in metabolomics. The simultaneous analysis of the exo-metabolome further increased our understanding, allowing the quantification of metabolites whose presence was only detected in the extracellular environment, and providing clues on whether the changes observed in the endo-metabolome may be due to different catabolic/anabolic rates or to altered partition across the plasma membrane.

**Figure 6:**
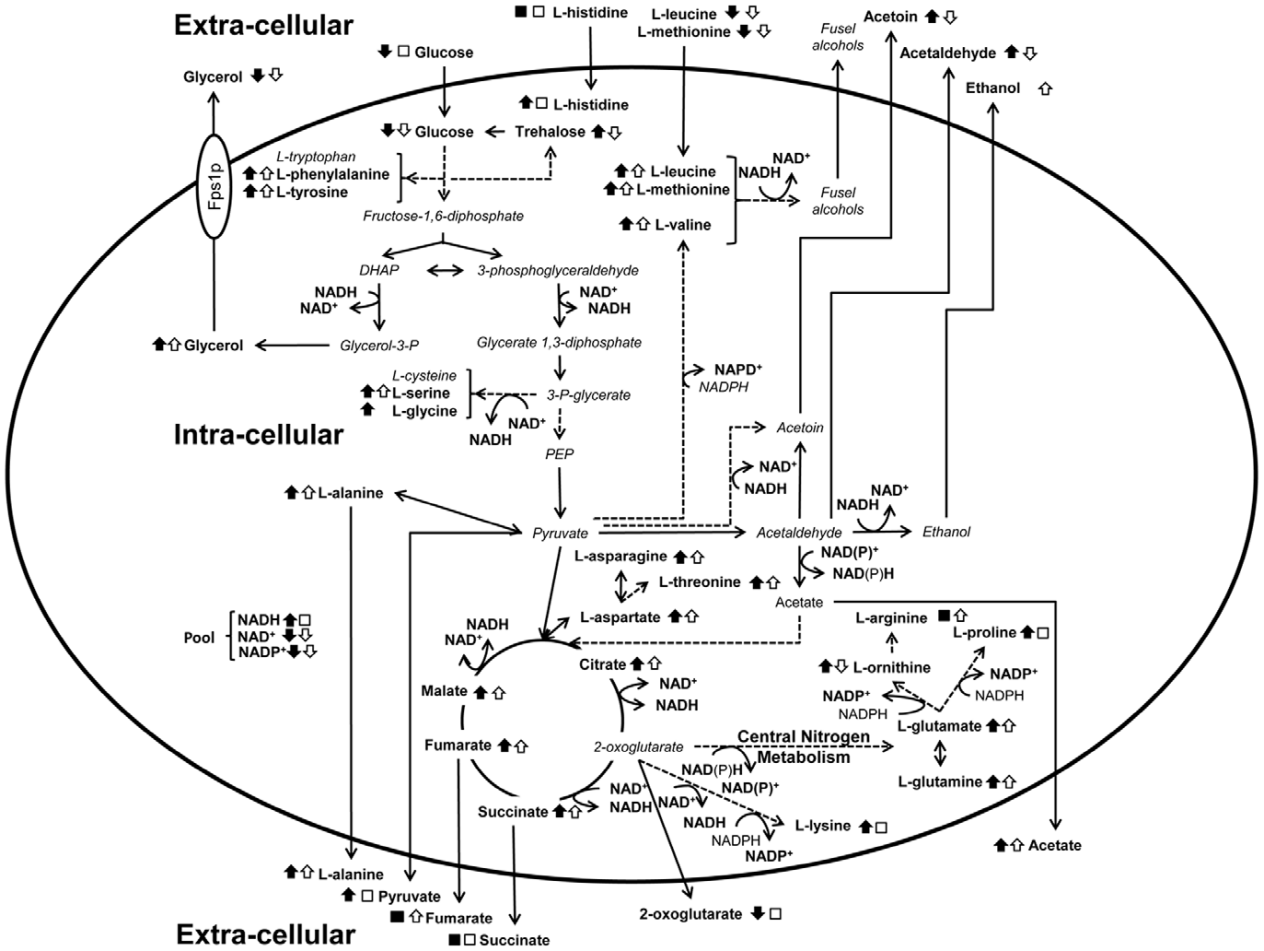
Integrative overview of the metabolic changes occurring under ethanol stress or upon *FPS1* deletion. Changes found to occur (as indicated by the black or white arrows) or not (□,▪) in yeast cells growing in the presence of inhibitory concentrations of ethanol (closed symbols), or upon *FPS1* deletion (open symbols) are shown. The metabolic scheme was based on information gathered in the KEGG PATHWAY Database (http://www.genome.jp/kegg/pathway.html) and in Yeast Biochemical Pathway Database (http://pathway.yeastgenome.org/).

The changes induced by ethanol stress at endo- and exo-metabolome level identified in this work are summarized in Figure 6. A number of metabolic effects from alcohol appear to be directly linked to the production of an excess of both NADH and acetaldehyde. Since ethanol exposure leads to this sort of reductive stress, the latter might trigger the induction of pathways aimed at the elimination of NADH excess (e.g. the conversion of pyruvic acid to lactic acid, the synthesis of glycerol or fatty acids, or the mitochondrial electron transport chain) and thus in the down-regulation of other metabolic pathways needed for optimal growth. NAD(P)^+^ and NAD(P)H, besides their role in determining the cell redox status [42], are critical determinants of *in vivo* reaction kinetics, being involved in over 200 reactions throughout yeast metabolism [43], [44]. It is therefore reasonable to hypothesize that the huge differences in the yeast metabolome in the presence of increasing ethanol concentrations are, at least partially, linked with the perturbation of the cell redox status. In fermentative or respiro-fermentative growth conditions, the NADH formed during glycolysis is reoxidized through the reduction of acetaldehyde to ethanol, maintaining in this way the intracellular redox balance [45]. However, Martini *et al.* [46] showed that the metabolic yield of ethanol production in *S. cerevisiae* decreases with increasing concentrations of exogenously added ethanol, in agreement with the known toxic effects of ethanol, namely, decreased fermentation productivity and ethanol yield [11], [47], [48], [49]. Therefore, and since the formation of glycolytic NADH beyond the cellular capacity for its reoxidation will lead to reduced conditions, an inhibition in the yeast fermentative capability, particularly at the level of the final alcohol dehydrogenase reaction, may be causing an imbalance in the cell redox status. Another possible cause for such an apparent decreased ability to regenerate NAD^+^ may be the inhibitory effect of ethanol at the level of the mitochondrial respiratory chain, whose function depends deeply on membrane integrity. Strongly linked with the intracellular redox status is the type and amount of by-products formed during yeast metabolism [44], [45], [50], [51], [52], [53]. Indeed, the exo-metabolome analysis carried out herein showed a higher accumulation in the broth medium of acetate, pyruvate, acetoin and acetaldehyde when ethanol is exogenously added, in a dose dependent way. The results obtained are also consistent with the previous observation, based on transcriptomic analysis, that ethanol challenge leads to a strong induction of the expression of *TDH1*, encoding a glyceraldehyde-3-phosphate dehydrogenase that utilises NAD^+^ as a cofactor, suggesting that ethanol stressed-cells may experience reductive stress [2], [22]. Also in agreement with this notion, the ratio of reduced to oxidized glutathione was observed, in this study, to increase in a dose-dependent manner in yeast cells growing exponentially in the presence of inhibitory ethanol concentrations.

Li *et al.* [34], [35] suggested that in the presence of ethanol stress there is an inhibition of glycolysis. The increased amount of intracellular and/or excreted glycolytic and TCA cycle intermediate metabolites, including pyruvate, citrate, malate, fumarate and succinate suggests an increase in overflow metabolism in turn of the pyruvate branch-point. The higher accumulation of pyruvate in the broth medium in the presence of ethanol stress is probably a consequence of an increase in difference between the fluxes upstream and downstream of pyruvate, which may be linked with the demand of energy supply for stress responsive processes. The referred overflow metabolism in turn of the pyruvate branch-point may underlie the changes registered in the intracellular content of some amino acids [37], [54]. Indeed, the current endo-metabolome analysis identified an overall increase in the intracellular content of most amino acids, which corroborates the observations made in previous metabolomic studies focused on the ethanol stress response [34], [35]. One general explanation that could account to an increase in the intracellular concentration of each amino acid could be an inhibition in protein synthesis in the presence of ethanol stress. However, this explanation apparently cannot account for the fact that not all the amino acids that were monitored increased their content with increasing ethanol concentrations as it is the case for L-arginine, L-lysine and L-ornithine. Moreover, the increase fold changes were found to vary significantly between the different amino acids monitored. In particular, the intracellular content of L-alanine and L-valine, two pyruvate-related amino acids, showed a dramatic accumulation in the exponential growth phase with increasing concentrations of ethanol, reinforcing the idea of overflow metabolism in turn of the pyruvate branch-point, as previously suggested to occur under other stress challenges, as was reported for very high gravity fermentation and in propionic acid stressed cells [37], [54]. The increased intracellular content of L-aspartate, L-asparagine and L-threonine was also shown to occur in yeast cells grown in the presence of increasing ethanol concentrations. These aminoacids are derived from oxaloacetate, an intermediate of tricarboxylic acid (TCA) pathway that occurs in the matrix of mitochondria. Similar trends were observed in the contents of the TCA pathway intermediates succinate, malate, citrate and fumarate. These observations are consistent with previous indications, suggesting that, even in the presence of glucose-repressing conditions, mitochondrial function is important for yeast tolerance against ethanol stress [16]. During alcoholic fermentation, the TCA pathway operates as two branches, a reductive and an oxidative branch [55], aiming the supply of C4 and C5 compounds (oxaloacetate and 2-oxoglutarate), the precursors of L-aspartate and L-glutamate, respectively [56]. Interestingly, the interconversion of L-glutamate and L-glutamine, whose concentrations were found to increase in yeast cells thriving in the presence of ethanol stress, has been implicated in the balance of the cell redox status [57], [58], [59], [60]. Since L-glutamate is formed from the condensation of ammonium and 2-oxoglutarate, the accumulation of lower amounts of 2-oxoglutarate in the broth medium in the presence of ethanol stress is consistent with the changes registered in the intracellular content of L-glutamate. Aromatic amino acid concentrations were also found to increase in the presence of increasing ethanol concentrations, which may correlate with the identification of aromatic amino acids biosynthetic enzymes as being required for maximal ethanol resistance [26]. Although with different magnitudes, we also observed the intracellular accumulation of the amino acids that were supplemented in the growth medium due to the yeast strain auxotrophies (L-leucine, L-methionine and L-histidine) in a dose dependent way. The accumulation of L-leucine and L-methionine may rely on the fact that they may be degraded into fusel alcohols, such as isoamyl alcohol or methionol, in a NAD^+^ regenerating reactions [50]. Interestingly, changes observed in the L-leucine content are consistent with a recent study suggesting that an enhanced uptake and/or utilization of L-leucine may be responsible for improved growth in the presence of ethanol [61]. Furthermore, these observations may also indicate the diffusion of externally supplied hydrophobic amino acids across the plasma membrane, which may occur as a consequence of membrane permeabilization by ethanol.

The increased accumulation of several metabolites known for their function as cellular stress protectants was also identified in yeast cells growing under ethanol stress. This was the case of L-proline and trehalose, whose accumulation in yeast cells [62], [63], together with the increased expression of the corresponding biosynthetic genes [16], [24], [26] had been previously implicated in cell protection against ethanol stress. Both L-proline and trehalose have been shown to enhance the stability of membranes, and to contribute to protect proteins from denaturation and aggregation (reviewed in [64]), two important features for cells growing in the presence of ethanol stress, which induces low water activity stress. In this work we show that L-proline and trehalose accumulation occurs in an ethanol-dose dependent manner in yeast cells growing exponentially in the presence of this stress.

This study also focused on the effect of *FPS1* expression in the yeast metabolome, as summarized in Fig. 6. Consistent with the decreased exponential growth rate registered upon *FPS1* deletion under control conditions, it was interesting to observe that, even in the absence of ethanol stress, there are significant differences at the metabolome level between the *fps1Δ* deletion mutant and the corresponding parental strain. The major change registered was the 13-fold increase in the intracellular accumulation of glycerol in the absence of Fps1, accompanied by a decrease in extracellular glycerol concentration, which is in agreement with the proposed role for this aquaglyceroporin in facilitating the diffusion of glycerol across the plasma membrane [28], [65]. Simultaneously, a decrease in NAD^+^ content was also observed in yeast cells devoid of *FPS1*, which may imply that glycerol-3-phosphate dehydrogenase activity, that catalyses a step required for NAD^+^ regeneration and leads to glycerol formation, is inhibited by the over-accumulation of glycerol. Indeed, *FPS1* deletion was previously shown to decrease not only glycerol transport, but also total glycerol production [65]. This result suggests that the *fps1Δ* deletion mutant may experience a permanent state of redox imbalance, similar to the one observed in this work to affect wild-type cells growing under ethanol stress. Consistent with the hypothesis raised herein that *fps1Δ* cells may experience reductive stress, Fps1 was previously identified as conferring resistance to the reductive stress inducers dithiothreitol and mercaptoethanol [66]. Given the obtained results, *fps1Δ* cells may exhibit an increased susceptibility to ethanol stress due to their intrinsic redox imbalance, which may be further exacerbated under ethanol stress. The higher ethanol tolerance exhibited by cells expressing *FPS1* [16] is hypothesized to be due to their increased ability to regenerate NAD^+^, through the production and release of glycerol [28], and to the increased trehalose levels produced, assuming that a link between trehalose and glycerol synthesis may exist to fine-tune the concentration of these two molecules with partially overlapping functions as osmoprotectants. Consistent with this hypothesis is the increased glycerol content found in wild-type cells growing exponentially in the presence of ethanol stress, except for those exposed to 6% ethanol, which instead exhibit a very high increase in trehalose levels.

The intra-cellular concentration of a number of amino acids, including L-tyrosine, L-phenylalanine, L-alanine, and L-glutamine, and TCA-related metabolites, including succinate, fumarate and citrate was found to be increased in the *fps1Δ* deletion mutant, when compared to the parental strain, mimicking ethanol stress repercussions exerted upon the parental strain. Particularly interesting is the observation that *fps1Δ* cells display a decreased concentration of trehalose both in control conditions and under ethanol stress. Given the importance of this metabolite for ethanol tolerance [64], the decreased ability of the *fps1Δ* deletion mutant to produce trehalose may also underlie its increased susceptibility towards ethanol stress.

### Concluding Remarks

Altogether, this study provides evidence that support the notion that ethanol stress induces reductive stress in yeast cells, which appears to be dealt with by the increase in the rate of NAD^+^ regenerating bioreactions. The action of Fps1 in mediating the passive diffusion of glycerol is highlighted as a key factor in the maintenance of redox balance, an important feature for ethanol stress resistance. The obtained results further reinforce the idea that metabolomic approaches may be crucial tools to understand the function of membrane transporters/porins, such as Fps1, particularly by providing clues on their physiological substrates and/or the effect of their expression at the global metabolism level. By providing a systems level view of the metabolome changes contributing to ethanol stress tolerance in yeast, this metabolic analysis appears as an important approach to guide the design of process conditions and of more robust yeast strains optimized to improve industrial fermentation performance.

## Supporting Information

Figure S1 **Representative endo- and exo-metabolome profiles.** High-resolution ^1^H NMR spectra from the endo- and the exo-metabolome profiles of *S. cerevisiae* BY4741 and the *fps1Δ* deletion mutant cells harvested during the exponential phase of growth (OD_600nm_ = 1.0) in the presence of different ethanol concentrations (% v/v). Spectra are representative of all the replicates obtained for each growth condition and were normalized to the reference TSP (δ = 0 ppm).
(TIF)

Figure S2 **Metabolite-metabolite correlations map.** Metabolite to metabolite correlations based from the BY4741 parental strain endo-metabolome in the presence of different ethanol concentrations (0, 2, 4 and 6% v/v). For each metabolite, a characteristic bin in the NMR spectrum was used. Metabolites were grouped based on the PCA loadings considering all principal components of the PCA model (dendrogram using ward clustering distance measure). Each square represents the correlation between the metabolite heading the column and the metabolite heading the row. Each square indicates a given R^2^ value (coefficient of determination) resulting from a Pearson correlation analysis in a false color scale (see color key at the bottom).
(TIF)

Table S1 **Metabolites identified in the endo-metabolome data.**
(XLSX)

Table S2 **Metabolites identified in the exo-metabolome data.**
(XLSX)

Table S3 **Endo-metabolome data.**
(XLSX)

Table S4 **Metabolite analysis of the endo-metabolome of BY4741 parental strain.**
(XLSX)

Table S5 **O-PLS-DA modelling of the endo-metabolome of BY4741 parental strain in the presence of different ethanol concentrations.**
(XLSX)

Table S6 **Exo-metabolome data.**
(XLSX)

Table S7 **Metabolite analysis of the exo-metabolome of BY4741 parental strain.**
(XLSX)

Table S8 **O-PLS-DA modelling of the endo-metabolome of the BY4741 parental strain and Dfps1 deletion mutant in the presence of different ethanol concentrations.**
(XLSX)

Table S9 **Metabolite analysis of the endo-metabolome of** ***fps1Δ*** **deletion mutant strain.**
(XLSX)

Table S10 **Metabolite analysis of the endo-metabolome of the parental strain and of the** ***fps1Δ*** **deletion mutant.**
(XLSX)

Table S11 **Metabolite analysis of the exo-metabolome of** ***fps1Δ*** **deletion mutant strain.**
(XLSX)

Table S12 **Metabolite analysis of the exo-metabolome of the parental strain and of the** ***fps1Δ*** **deletion mutant.**
(XLSX)
