# Supplementary figures and images for: Quantitative 1H-NMR-Metabolomics Reveals Extensive Metabolic Reprogramming and the Effect of the Aquaglyceroporin FPS1 in Ethanol-Stressed Yeast Cells
Source: PLoS One. 2013 Feb 8;8(2):e55439. doi: 10.1371/journal.pone.0055439 (PMC3568136; doi:10.1371/journal.pone.0055439)

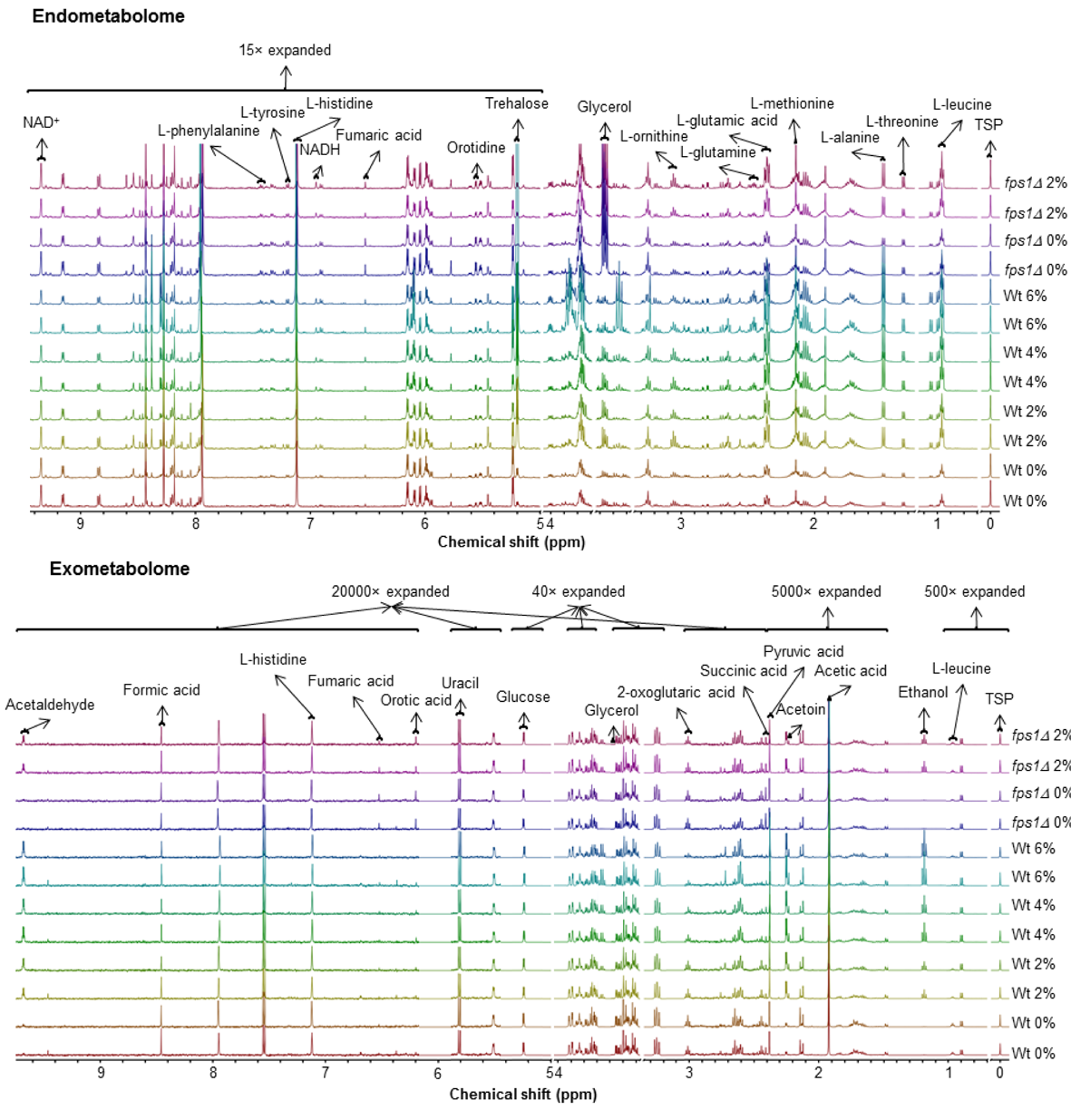

Supplement: Figure S1 — Representative endo- and exo-metabolome profiles. High-resolution 1H NMR spectra from the endo- and the exo-metabolome profiles of S. cerevisiae BY4741 and the fps1Δ deletion mutant cells harvested during the exponential phase of growth (OD600nm = 1.0) in the presence of different ethanol concentrations (% v/v). Spectra are representative of all the replicates obtained for each growth condition and were normalized to the reference TSP (δ = 0 ppm). (TIF) [file pone.0055439.s001.tif]

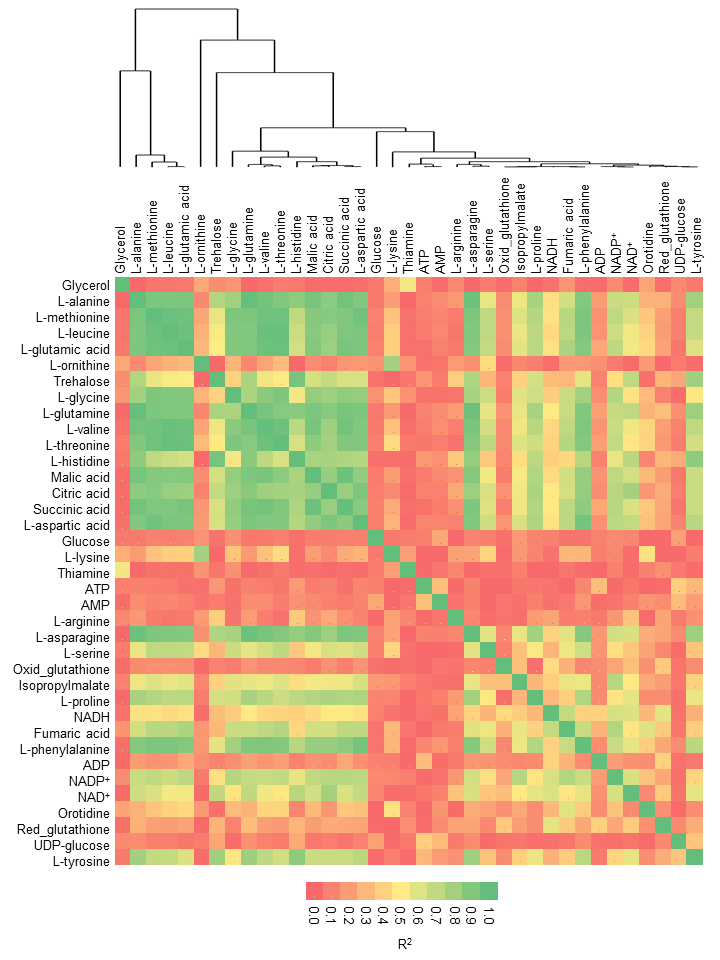

Supplement: Figure S2 — Metabolite-metabolite correlations map. Metabolite to metabolite correlations based from the BY4741 parental strain endo-metabolome in the presence of different ethanol concentrations (0, 2, 4 and 6% v/v). For each metabolite, a characteristic bin in the NMR spectrum was used. Metabolites were grouped based on the PCA loadings considering all principal components of the PCA model (dendrogram using ward clustering distance measure). Each square represents the correlation between the metabolite heading the column and the metabolite heading the row. Each square indicates a given R2 value (coefficient of determination) resulting from a Pearson correlation analysis in a false color scale (see color key at the bottom). (TIF) [file pone.0055439.s002.tif]
